# Regioselective Palmitoylation of 9-(2,3-Dihydroxy- propyl)adenine Catalyzed by a Glycopolymer-enzyme Conjugate

**DOI:** 10.3390/molecules21050648

**Published:** 2016-05-16

**Authors:** Jana Brabcová, Jiří Blažek, Marcela Krečmerová, Jiří Vondrášek, Jose M. Palomo, Marie Zarevúcka

**Affiliations:** 1 Institute of Organic Chemistry and Biochemistry, AS CR, Flemingovo nám. 2, Prague 6, Czech Republic; brabcova@uochb.cas.cz (J.B.); blazek@uochb.cas.cz (J.B.); krecmerova@uochb.cas.cz (M.K.); vondrasek@uochb.cas.cz (J.V.); 2 Departamento de Biocatálisis, Instituto de Catálisis (CSIC) Campus UAM Cantoblanco, Madrid 28049, Spain

**Keywords:** regioselectivity, palmitoylation, glycosylation, chemical modification

## Abstract

The enzymatic regioselective monopalmitoylation of racemic 9-(2,3-dihydroxypropyl)- adenine (DHPA), an approved antiviral agent, has been performed by an immobilized form of *Candida antarctica* B lipase (CAL-B) using a 4:1 DMF/hexane mixture as the reaction medium. To improve the chemical yield of the desired monopalmitoylation reaction, solid-phase chemical modifications of the lipase were evaluated. The reaction yield was successfully increased obtaining 100% product after a second treatment of the product solution with fresh immobilised chemically glycosylated-CAL-B.

## 1. Introduction

**Figure 1:**
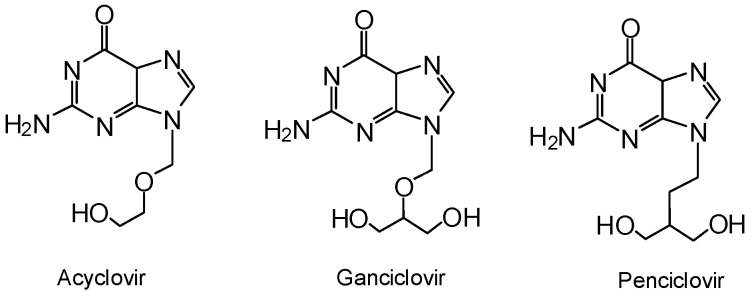
Acyclic nucleoside antiviral agents.

Acyclic nucleoside analogues are a class of molecules demonstrating antiviral activity [1,2,3,4,5]. Several representatives of this group such as acyclovir, ganciclovir or penciclovir (Figure 1) are approved antiviral agents with activity targeted against herpes viruses. A promising candidate is 9-(2,3-dihydroxypropyl)adenine (DHPA), which has shown antiviral potency by the inhibition of *S*-adenosylhomocysteine (SAH) hydrolase [6,7]. Indeed, this molecule is an approved drug for the topical treatment of herpes labialis (HSV-1) in the former Czechoslovakia, marketed under the name Duvira^®^ gel. However, the low oral bioavailability of all acyclic nucleoside analogues is a limitation for their use as topical drugs only.

The modification of the drugs by hydrophobic moieties has been established as a successful strategy for improving their bioavailability and pharmacological properties [8,9,10,11,12]. Among the different hydrophobic groups available, the introduction of a palmitoyl moiety might be an advantage, because it is one of the lipids constituting the plasmatic membrane. Palmitic acid has been shown as a typical group for example for the membrane binding and membrane targeting of lipidated proteins [13,14]. In this way, the introduction of only one hydrophobic moiety by a specific and regioselective way would be mandatory to improve drug bioavailability. For that purpose, the application of a green biocatalytic approach seems to be an excellent alternative.

Lipases are popular enzymes utilized for esterification reactions in organic synthesis [15,16,17]. Although they are highly versatile, specificity or regioselectivity are sometimes not expected. Therefore, the application of immobilization techniques [18,19,20] or chemical modification approaches [21,22,23,24,25,26] has chemically modified lipases, especially after the protein glycosylation process on the solid phase made it possible to overcome these drawbacks, obtaining exceptional improvements of catalytic properties, giving excellent enantio- or regioselective catalysts for example in carbohydrate or nucleoside deprotection [27,28], the acylation of cholesterol analogues [29] or the desymmetrisation of prochiral molecules [30].

Here we present the enzymatic palmitoylation of DHPA catalysed by immobilised lipases. Moreover, the cytotoxicity and antiviral activity of the *S*-enantiomer of palmitoylated DHPA in different cell cultures were evaluated.

## 2. Results and Discussion

**Scheme 1:**
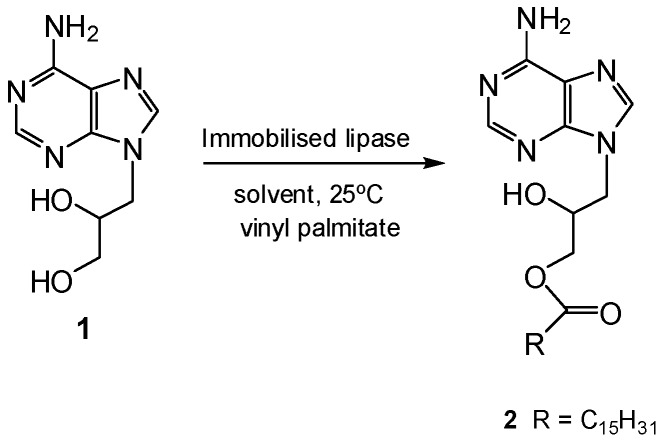
Enzymatic esterification of DHPA.

First, a screening of different lipases (*Candida antarctica*, *Pseudomonas fluorescens*, *Bacillus thermocatenulatus* and *Thermomyces lanuginosus)* soluble and in immobilized form (Lewatit 1600, octadecyl-Sepabeads or Purolite) for a suitable candidate for the enzymatic palmitoylation of DHPA (Scheme 1) was evaluated. *Candida antarctica* lipase B immobilized on Lewatit 1600 resin (Lew-CAL-B) was the only biocatalyst active in the esterification in neat DMF, although low conversion was noticed (entry 1, Table 1), demonstrating the difficulty of this enzymatic process.

Thus, the biotransformation was performed using different DMF-solvent combinations, considering our experience with the solubility of the DHPA in water and specially the ester derivative [31].

To address this issue, the addition of hexane improved the palmitoylation yield, with 6.4% after 24 h using 20% (*v*/*v*) hexane in the final volume mixture (entry 2, Table 1). The addition of 2-methyl-2-butanol in the same proportion also gave positive results but the presence of pyridine or ionic liquids in the solvent mixture produced a negative result for the esterification capacity of the biocatalyst (Table 1).

**Figure 2:**
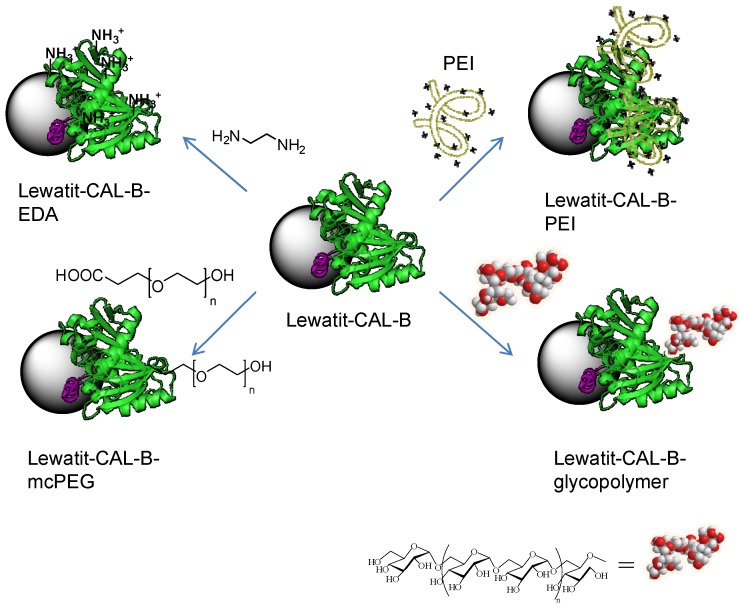
Different chemical modification of Lewatit-CAL-B on the solid phase.

Thus different chemical modification strategies (by non-covalent or covalent attachment of molecules to the protein) were then applied to the Lew-CAL-B preparation (Figure 2). One strategy was based on the coating of the protein surface of the immobilized enzyme by using polyethylenimine (PEI) (Lew-CAL-B-*PEI*). Another strategy was the modification of the carboxylic groups of residues in Asp and Glu amino acids in the protein by covalent modification using carbodiimide as the activating agent (Lew-CAL-B-*EDA*). Finally two specific strategies were then applied to modify the *N*-terminus of the protein. A monocarboxylated polyethylene (mcPEG1500) and a glycosylated dextran polymer (D1500) were used obtaining the respective heterogeneous semisynthetic enzymes (Lew-CAL-B-*mc-PEG* and Lew-CAL-B-*D1500*).

These new catalysts were used in the monopalmitoylation of **1** under the optimal conditions after 24 h (Table 2). The Lew-CAL-B-*EDA* preparation exhibited 50% less active when compared with the non-modified biocatalyst. This denotes the possibility of the coating with PEI and the chemical modification with the PEG (Lew-CAL-B-PEI and Lew-CAL-B-mc-PEG preparations) improved the enzyme activity by three-fold and still produced the palmitoylated **2** in moderate yield.

However, the site-specific glycosylation of the *N*-terminus of CAL-B by the glypolymer D1500 enhanced the synthetic activity towards the production of **2** with almost 50% yield (almost 10 times higher when compared to the non-modified one). Moreover a full conversion (>99%) of **2** was obtained after the second treatment of the product solution with the heterogeneous semisynthetic enzyme (Lew-CAL-B-*D1500*). Also the site-selective glycosylation was carried out or proceeded by using other activated bigger-size polymers however no improvements were found.

**Figure 3:**
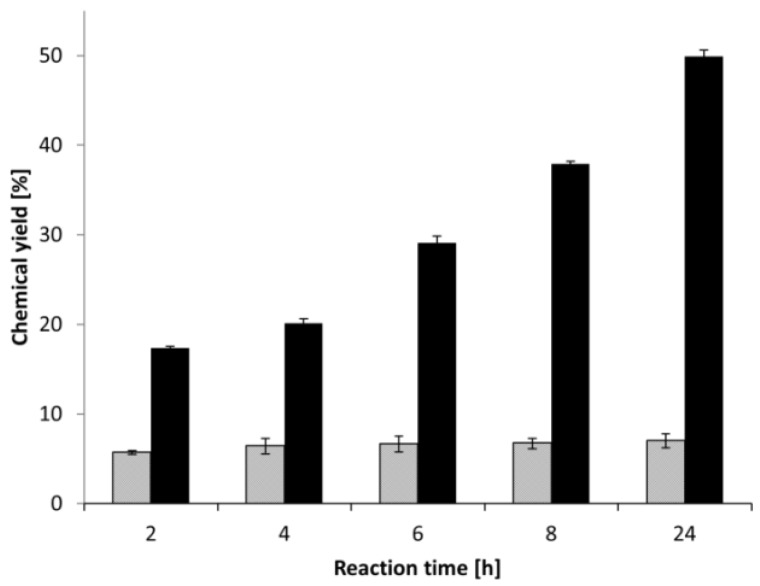
The reaction course of DHPA palmitoylation catalysed by Lewatit-CAL-B catalyst. Lewatit-CAL-B (**gray**), glycosylated Lewatit-CAL-B (**black**).

The reaction course (Figure 3) has demonstrated that the conversion stopped after 2 h for Lewatit-CAL-B, whereas the specific chemical glycosylation allowed the conversion to continue from 18% at 2 h to 50% at 24 h, after which (at 48 h) the reaction was not significantly changed. Therefore, the glycosylation of the enzyme helped to reduce the substrate inhibition and increased the conversion progress successfully.

**Figure 4:**
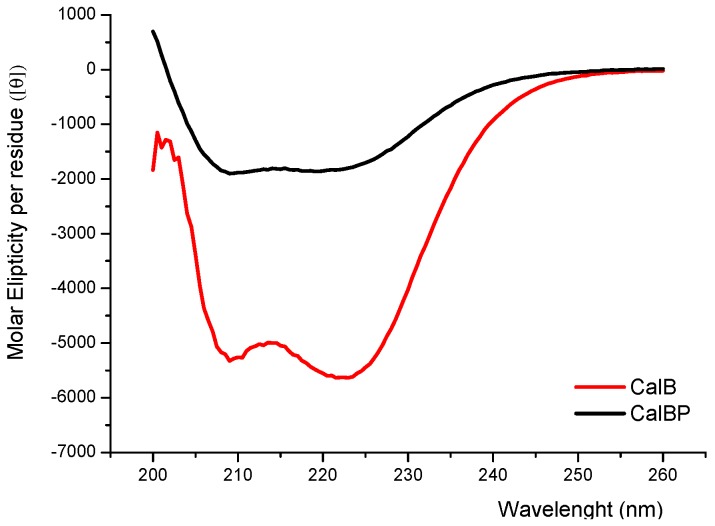
Circular dichroism spectra of CAL-B enzymes. CAL-B (**red**), glycopolymer-CAL-B (**black**).

Furthermore the incorporation of the glycopolymer in the *N*-terminal position of the enzyme caused modifications in its secondary structure as confirmed by circular dichroism; particularly, the content of alpha-helix was reduced (Figure 4). These structural variations might explain the increase of conversion progress, which is caused by the better favouring of the substrates into the active site of CAL-B.

**Figure 5:**
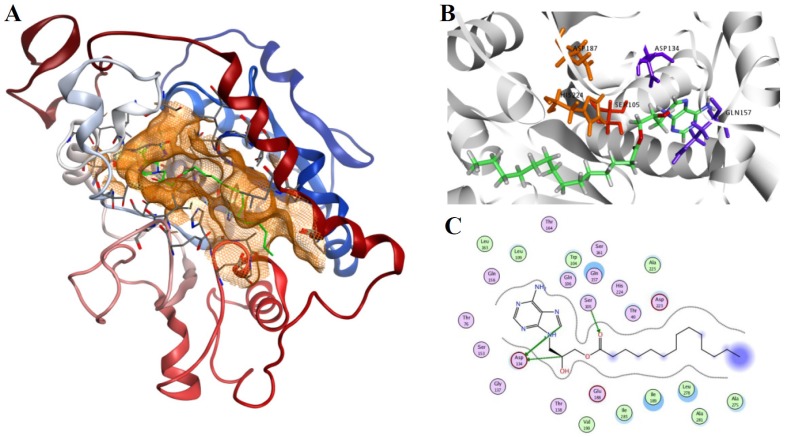
(**A**) The best model of the product 3-(6-amino-9*H*-purin-9-yl)-2-hydroxypropyl palmitate (**2**) in the active site of CAL-B. This most productive conformation situates the adenine in the large hydrophobic pocket due to a better enzyme–substrate interaction; (**B**) a detail of **2** (represented as green sticks) placement in the binding cavity of the lipase, the catalytic triad Asp-His-Ser is shown as orange sticks (Ser105 is red); (**C**) a schematic view of potential hydrogen-bond stabilisation appearing between: (i) Ser105 and the palmitoyl group; (ii) Asp134 and the 9′*H*-purin of the adenine ring and 2-OH of propyl. The hydrogen bonds between ligand atoms and amino acids of the active site are shown as dotted lines. Crystal structures with pdb code 1LBS and 1LPP were used.

The binding mode of product **2** in the active site of the 1LBS was analyzed based on the result of the docking procedure performed by the LigX module. The final position of the ligand within the cavity provides the anatomy of the binding pocket and the amino acids participating in the interactions (Figure 5). Most of the non-covalent interactions are realized through a dispersion interaction of the aliphatic part of the ligand, with the amino acids Ser105, Gln157 and Asp134 participating in the binding (Figure 5).

The *S*-enantiomer of DHPA has been demonstrated to exhibit interesting antiviral activity [7]. In order to obtain a compound with better bioavailability, we synthesised the palmitoylated (S)-DHPA following a previously described procedure and its biological activity was evaluated with several viruses—Para-influenza-3 virus, Reovirus-1, Sindbis virus, Coxsackie virus B4, Punta Toro virus, Vesicular stomatitis virus and Respiratory syncytial virus (data not shown). Although the concentration exhibiting biological activity was low as compared to commercial antivirals tested, the addition of the palmitoyl group increased the antiviral activity. There was a small but consistent difference from the parent analogue. The antiviral activity of 2 (>100 µg/mL) was 2.5 times higher than those of the parent compound (>250 µg/mL) for all seven types of viruses studied. This led to a hypothesis that the parent compound DHPA (**1**) must either be more efficiently released from its palmitoylated DHPA (**2**) prodrug molecule or better transported to the cells, or both. The in vivo bioavailability will be the subject for further study.

## 3. Materials and Methods

### 3.1. Materials

Dextran (Mr 1500 Da), polyethyleneglycol (PEG) (Mr 1500 Da), polyethylenimine (Mr 1200), ethylenediamine, *p*-nitrophenyl butyrate (*p*NPB), 1-ethyl-3-(3-dimethylaminopropyl)-carbodiimide (EDC), ethylenediamine (EDA) were from Sigma Aldrich (Prague, Czech Republic). *Candida antarctica* B lipase (CAL-B) was kindly donated by Novozymes (Bagsvaerd, Denmark). Octadecyl-Sepabeads was kindly donated by Resindion Srl. (Tokio, Japan). Lewatit1600 was from Lanxess (Cologne, Germany) and Purolite A503 was from Purolite (Barcelona, Spain). Aldehyde-activated dextran (glycopolymer D1500) and monocarboxylated PEG1500 were prepared as previously described [22]. (*S*)-9-(2,3-dihydroxypropyl)adenine (DHPA) was prepared as previously described [31]. All organic solvents and other reagents were of analytical grade.

### 3.2. Enzyme Activity Assay

In order to study the immobilisation process, the activities of the soluble and immobilised lipase catalysts were analysed spectrophotometrically measuring the increment in absorbance at 348 nm produced by the release of *p*-nitrophenol (*p*NP) (∈ = 50 M^−1^∙cm^−1^) in the hydrolysis of 0.4 mM of *p*NPB in 25 mM of sodium phosphate at pH 7 and 25 °C. To initiate the reaction, 0.05–0.2 mL of lipase solution or suspension were added to 2.5 mL of substrate solution. Enzymatic activity is given as a micromole of hydrolysed *p*NPB per minute per milligram of enzyme (IU) under the conditions described above.

### 3.3. Lipase Immobilisation

Commercial extract of CAL-B (4 mL, 4.5 mg protein/mL measured by Bradford) was dissolved in 10 mM sodium phosphate buffer at pH 7 (16 mL). One gram of octadecyl-Sepabeads, Lewatit 1600 or Purolite was added to this solution and the mixture was incubated at 28 °C for 3 h. The activity of the suspension and supernatant was assayed by the *p*NPB assay described below. The suspension was washed with distilled water (10 times with 100 mL). In all cases the immobilisation yield obtained was more than 95%. Finally, the immobilised lipases were dried by incubation in an oven at 37 °C for 2 h before their use in the transesterification of DHPA.

### 3.4. Chemical Modification of Immobilised Lewatit-CAL-B Preparation

A site-specific modification with glycopolymer: 10 mL of 10% oxidised (aldehyde) dextran representing Mw of 1500 (glycopolymer D1500) (33 mg∙mL^−1^) in 100 mM of a sodium phosphate buffer (pH 7.5) were added to one gram of immobilised Lewatit-CAL-B preparation. After 36 h of gentle stirring, sodium borohydride (1 mg/mL) was added, followed by another addition of sodium borohydride after 15 min. Thirty min later, the mixture was filtered and washed abundantly with water.

A site-specific modification with monocarboxylated polyethylene glycol: 1-Ethyl-3-(3-dimethylaminopropyl)carbodiimide (EDC, 50 mmol) and 8.2 µmol of dimethylamine pyridine were added to a solution of monocarboxylated polyethylene glycol *(mc-PEG)* Mw = 1500 (33 mg∙mL^−1^) in 5 mM of a sodium phosphate buffer (pH 7). One gram of biocatalyst was added to 20 mL of this solution, and the reaction was maintained for 48 h. Then, the preparation was filtrated and washed abundantly with distilled water.

Chemical amination: One gram of biocatalyst was added to 10 mL of a solution of 1 M of EDA. Then, solid EDC (10 mM) was added and the pH was adjusted to 4.75. The preparation was incubated for 2 h and then washed twice with distilled water and once with 5 mM of phosphate buffer (pH 7).

Coating with polyethyleneimine: one gram of polyethyleneimine (PEI) was dissolved at 100 mL solution of water/dioxane (7/3, *v*/*v*) at pH 5. One gram of immobilised biocatalyst was added to this solution and then solid EDC (10 mM) was added.

The suspension was gently stirred for 1 h and the modified enzyme preparations were abundantly washed with distilled water.

### 3.5. Transesterification Reactions

Palmitoylation: 50 mg of immobilised enzyme were added to 375 µL of a solution consisting of DHPA (1.6 mM) and different ratios of solvent:cosolvent and vinyl palmitate (from 1 to 10 eq). The catalyst was dried at 40 °C overnight and washed before reaction by DMF/hexane (4/1, *v*/*v*). Molecular sieves were added into each reaction mixture (40 mg per 375 µL reaction mixture). The reaction was performed in Eppendorf tubes under constant stirring at 25 °C and the progress of the reaction was monitored by TLC analysis. The final stage consisted of the filtration off of the enzyme and the evaporation of the solvent. The reaction mixture was dissolved in the mixture chloroform:methanol (4:1) and measured by HPLC.

### 3.6. Analytical Methods

The ^1^H-NMR spectra were recorded on a UNITY 500 spectrometer (in a FT mode, Varian) at the respective 499.8 and 125.7 MHz frequency values either in deuteriochloroform using tetramethylsilane (δ = 0) as an internal reference or in hexadeuteroacetone using the central line of the solvent (δ = 2.13) as an internal reference. TLC was carried out on precoated silica gel TLC plates. A column (250 mm ×4 mm) filled with a Separon SGX C_18_ solid phase (5 μm; Watrex, Prague, Czech Republic) was employed for the HPLC analysis of the sample composition using the following gradient of Solvent A (water) and Solvent B (acetonitrile): from 100% to 0% of Solvent A in 30 min as a mobile phase at 1 mL∙min^−1^. During the HPLC analysis, the compounds were detected at 254 nm.

#### Preparative TLC

The products of the enzymatic reactions (the remaining DHPA, **2**) were separated by preparative TLC (using CHCl_3_:CH_3_OH (4:1)) and characterised by ^1^H-NMR and ^13^C-NMR analysis.

### 3.7. 3-(6-Amino-9H-purin-9-yl)-2-hydroxypropyl palmitate (**2**)

^1^H-NMR (DMSO-*d*_6_): 8.11 (1H, s, H-2), 8.04 (1H, s, H-8), 7.19 (2H, bs, -NH_2_), 5.48 (1H, d, *J*_OH-2’_ = 5.0 Hz, -OH), 4.23 (1H, m, H-1’b), 4.06–4.14 (2H, m, H-1’a, H-2’), 3.96 (1H, bdd, *J*_gem_ = 11.4, *J*_3’b-2’_ = 4.8, H-3’b), 3.93 (1H, bdd, *J*_gem_ = 11.4 Hz, *J*_3’a-2’_ = 5.3, H-3’a), 2.27 (2H, t, *J*_5’-6’_ = 7.4 Hz, H-5’), 1.50 (2H, pent, *J*_6’-5’_ = 7.3 Hz, H-6’), 1.17–1.31 (12H, m, H-7’-12’), 0.84 (3H, t, *J*_19’-18’_ = 7.0 Hz, H-13’). ^13^C-NMR (DMSO–*d*_6_): 173.0 (C-4’), 156.1 (C-6), 152.5 (C-2), 149.9 (C-4), 141.7 (C-8), 118.8 (C-5), 66.7 (C-2’), 65.7 (C-3’), 46.3 (C-1’), 33.6 (C-5’), 31.5 (C-11’), 28.7–29.1 (C-7’-10’), 24.6 (C-6’), 22.3 (C-12’), 14.2 (C-13’). MS (ESI): [M + H]^+^ = 447.32, found 448.60.

### 3.8. Circular Dichroism and Fluorescence Spectroscopy

Circular dichroism (CD) spectra of the CAL-B and glycopolymer-CAL-B conjugate were recorded in a Chirascan spectropolarimeter (Applied Photophysics, Leatherhead, Surrey, UK) at 25 (±1) °C. Near-UV spectra were recorded at wavelengths between 250 and 310 nm in a 1 cm path-length cuvette, with 10 µM protein solutions in phosphate buffered saline, pH 7.2 (PBS; bioMerieux, Prague, Czech Republic). The far-UV spectra were measured at wavelengths between 190 and 250 nm in a 1 mm path-length cuvette, with 2 µM protein solutions in the same buffer. Blank measurements were made with the appropriate buffer. Fluorescence measurements were performed in a Varian Cary Eclipse Fluorescence Spectrophotometer (Agilent Technologies, Santa Clara, CA, USA) monitoring the intrinsic tryptophan fluorescence in the different proteins, using an excitation wavelength of 280 nm, with excitation and emission bandwidths of 5 nm, and recording fluorescence emission spectra between 300 and 400 nm. All spectroscopic measurements were made in water.

### 3.9. Docking Experiments

A 3D model of lipase with a ligand complex was constructed based on an X-ray structure of lipase B from *Candida antarctica* (1LBS PDBid). For docking and orientation of the ligand in the binding cavity, we utilised the similarity with an aliphatic ligand bound to *Candida rugosa* lipase (1LPP PDBid). The ligand conformation was refined by applying the LigX module of the MOE for the optimisation procedure and its final binding mode was selected by the best-fit model based on the London dG scoring function and the generalised Born method. Molecular images were generated with UCSF Chimera (http://www.cgl.ucsf.edu/chimera/).

## 4. Conclusions

In summary, a successful regioselective monopalmitoylation of 9-(2,3-dihydroxypropyl)- adenine has been described by using an immobilized form of a chemical glycosylated CAL-B lipase. The final product exhibited better antiviral activity than DHPA representing interesting target for future studies.

## Figures and Tables

**Table 1:** The effect of solvent in the palmitoylation of DHPA catalysed by Lew-CAL-B.

Entry	Solvent	Conversion 2 (%) ^a^
1	DMF	2
2	DMF: Hexane (4:1)	6.4
3	DMF: Hexane (3:2)	5.9
4	DMF: Ionic liquid ^b^ (4:1)	0.6
5	DMF: 2-methyl-2-butanol (4:1)	6.2
6	DMF:Pyridine (4:1)	1.1

^a^ conversion after 24 h measured by HPLC. The reaction conditions were: 1.6 mM DHPA, vinyl palmitate (1:10) and 50 mg of Lewatit-CAL-B (130 U/g); ^b^ 1-butyl-3-methylimidazolium tetrafluoroborate.

**Table 2:** Synthesis of monopalmitoylated DHPA catalysed by different chemically modified derivatives of the Lew-CAL-B ^a^.

Entry	Modified Biocatalysts	Conversion 2 (%)
1	-	6.2
2	Lew-CAL-B-*EDA*	3.5
3	Lew-CAL-B*-PEI*	16.2
4	Lew-CAL-B-*mc-PEG*	19.4
5	Lew-CAL-B-*D1500*	49.9 (>99) ^b^

^a^ conversion after 24 h. The reactions conditions were: 1.6 mM DHPA, vinyl palmitate (1:10) in DMF:hexane (4:1) and 50 mg of biocatalyst; ^b^ >99% conversion of palmitoylation was found after second treatment with the biocatalyst.
